# Genetic and Functional Characterization of a Conjugative KpVP-2-Type Virulence Plasmid From a Clinical *Klebsiella pneumoniae* Strain

**DOI:** 10.3389/fmicb.2022.914884

**Published:** 2022-07-22

**Authors:** Xuemei Yang, Xiaoxuan Liu, Yating Xu, Chen Yang, Edward Wai-Chi Chan, Hoi-ping Shum, Sheng Chen

**Affiliations:** ^1^Department of Infectious Diseases and Public Health, Jockey Club College of Veterinary Medicine and Life Sciences, City University of Hong Kong, Kowloon, Hong Kong SAR, China; ^2^State Key Lab of Chemical Biology and Drug Discovery, Department of Applied Biology and Chemical Technology, The Hong Kong Polytechnic University, Hung Hom, Hong Kong SAR, China; ^3^Department of Intensive Care, Pamela Youde Nethersole Eastern Hospital, Chai Wan, Hong Kong SAR, China

**Keywords:** *Klebsiella pneumoniae*, *Klebsiella pneumoniae* virulence plasmids type 2 (KpVP-2), conjugative plasmid, mobile genetic element, virulence

## Abstract

The main mechanism of virulence in *Klebsiella pneumoniae* is the acquisition of *K. pneumoniae* virulence plasmids (KpVPs), which include two dominant types, namely, KpVP-1 (carrying *iuc*1, *iro*1, *rmpA*, and *rmpA2*) and KpVP-2 (carrying *iuc*2, *iro*2, and *rmpA*). Both are non-conjugative and associated with different hypervirulent clones. In contrast to KpVP-1 reported in K1, K2, and other serotypes of *K. pneumoniae*, KpVP-2 was only reported in K2 strains and rarely characterized. In this study, we identified a conjugative KpVP-2-type virulence plasmid from a clinical hypervirulent *K. pneumoniae* strain. This plasmid was generated by the integration of conjugative transfer genes into the KpVP-2-type plasmid Kp52.145 II and could be readily conjugated to *Escherichia coli* strain EC600 and *K. pneumoniae* strains of various types which are clinically existing, mediating hypervirulence. Furthermore, this kind of conjugative KpVP-2-type virulence plasmid has been disseminated in clinical settings in Hong Kong and other regions of the world. The generation of conjugative virulence plasmid may promote its transmission and explain the evolution of this type of virulence plasmid.

## Introduction

*Klebsiella pneumoniae* is a human commensal and opportunistic pathogen that can cause severe hospital-acquired infections such as septicemia, pneumonia, urinary tract infections (UTI), and soft tissue infections, especially among patients with a compromised immune system (Li et al., 2014; Clegg and Murphy, 2016). However, over the last few decades, strains derived from classic *K. pneumoniae* (cKP) that have acquired additional genetic traits have emerged and made the infections caused by this strain more serious (Martin and Bachman, 2018). These genetic traits include antibiotic-resistant genes and virulence factors to make the stains either antibiotic-resistant or hypervirulent (Bialek-Davenet et al., 2014; Holt et al., 2015). *K. pneumoniae* employs a variety of virulence factors, such as capsule polysaccharides, siderophores, lipopolysaccharides, and fimbriae, to evade and inhibit the host immune response, colonize in the host, and obtain nutrition from the host (Shon et al., 2013). Hypervirulent *K. pneumoniae* (hvKP) can cause serious, life-threatening community-acquired infections in young and relatively healthy individuals, which is typically associated with the presence of a combination of multiple acquired virulence factors (Patel et al., 2014; Struve et al., 2015).

Apart from one *iro* lineage (*iro*3) and *rmpA* located in ICE*Kp1* with *ybt*, the main dispersal mechanism of acquired virulence factors is through plasmids, typically large IncFIB_K_ replicons known as *K. pneumoniae* virulence plasmids (KpVPs) (Lin et al., 2008; Lam et al., 2018). Two dominant types, namely, KpVP-1 (carrying *iuc*1, *iro*1, *rmpA*, and *rmpA2*) and KpVP-2 (carrying *iuc*2, *iro*2, and *rmpA*), which account for 74% and 14% of *K. pneumoniae* genomes carrying *iuc* and *iro*, respectively, have been characterized and are associated with different hypervirulent clones (Lam et al., 2018). Besides, KpVP-2-type virulence plasmids also do not carry loci encoding heavy metal resistances including copper, silver, and tellurite, which are highly conserved in KpVP-1 plasmids. KpVP-1, represented by pK2044, is mainly present in CG23, CG86, and CG65, whereas KpVP-2, represented by Kp52.145 II, is mainly present in CG380 and CG66 (Wu et al., 2009; Lery et al., 2014). The maintenance of KpVP-2 in specific hypervirulent clones through clonal expansion might be contributed by the lack of the conjugation machinery. However, KpVP-2 at low prevalence in other lineages suggests the possibility of dissemination by other transfer mechanisms (Lam et al., 2018). Even though the prevalence of KpVP-2 is much lower than that of KpVP-1, its influence might be largely underestimated. Since its emergence in or before 1935, ST66-K2 HvKP carrying KpVP-2-type virulence plasmids has been disseminated worldwide, causing serious human infections (Rodrigues et al., 2020; Kamau et al., 2021; Klaper et al., 2021).

The dominant virulence plasmid KpVP-1 is also non-conjugative. However, recent studies indicate that it can be mobilized between bacterial cells with the help of other conjugative plasmids (Xie et al., 2020; Xu et al., 2021) and also by generating mosaic plasmids comprising regions of KpVP-1 fused with those of conjugative plasmids, resulting in conjugative virulence plasmids (Yang et al., 2019). In contrast, the transmission of KpVP-2-type virulence plasmids has been rarely reported, and the mechanisms underlying the dissemination of this kind of plasmid remains unknown. In this study, we reported a KpVP-2-type virulence plasmid, carrying conjugative elements, which could be readily conjugated to *E. coli* and *K. pneumoniae* strains. Our data enriched the transmission mechanisms of virulence profiles in *K. pneumoniae* strains and alerted actions to prevent the spread of this kind of conjugative virulence plasmid.

## Materials and Methods

### Bacterial Strains and Identification

*Klebsiella pneumoniae* strains were isolated from clinical patients in hospitals located in Hong Kong SAR and mainland China, identified by the Vitek 2 system (bioMérieux, France), and confirmed by the matrix-assisted laser desorption/ionization–time-of-flight mass spectrometry apparatus (MALDI-TOF MS) (Bruker, Germany). A string test was performed on blood agar as previously described (Shon and Russo, 2012; Gu et al., 2018). Antimicrobial susceptibility testing was performed by the microdilution method. *E. coli* strain ATCC 25922 served as a quality control strain for susceptibility testing. Antimicrobial agents including ampicillin, ceftazidime, cefotaxime, meropenem, gentamicin, amikacin, azithromycin, ciprofloxacin, chloramphenicol, tetracycline, tigecycline, colistin, and spectinomycin were tested. All tests were performed in duplicate, and each test included three biological replicates per strain. The susceptibility was interpreted according to both Performance Standards for Antimicrobial Susceptibility Testing by the Clinical and Laboratory Standards Institute (CLSI) of 2021 (CLSI, 2021) and Clinical Breakpoints and Guidance by the European Committee on Antimicrobial Susceptibility Testing (EUCAST) of 2021 (Breakpoint tables for interpretation of MICs and zone diameters, 2021).

### DNA Sequencing and Bioinformatics Analysis

Genomic DNA was extracted using the PureLink™ Genomic Plant DNA Purification Kit for bacteria (Invitrogen, USA). The extracted DNA was sequenced *via* both the 150-bp paired-end Illumina NextSeq 500 platform (Illumina, San Diego, CA) and the long-read MinION platform (Oxford Nanopore Technologies, Oxford, UK). The Illumina libraries were prepared by the NEBNext Ultra II DNA Library Prep Kit for Illumina (New England Biolabs, USA). The MinION libraries were prepared using the SQK-RBK004 Nanopore Sequencing Kit and sequenced using the R9. 4.1 MinION flow cell. Both short and long reads were *de novo* hybrid assembled using Unicycler v0.4.8 (Wick et al., 2017). The assembled genome sequences were annotated with RAST v2.0 (Brettin et al., 2015). Multi-locus sequence typing (MLST) was determined by the Kleborate software based on the types of genetic variations in the seven housekeeping genes (Lam et al., 2021). Capsular typing on the assembled sequences was performed using Kaptive (Wyres et al., 2016). Virulence genes were identified by searching against the BIGSdb *Klebsiella* genome database (Jolley et al., 2018). Resistance genes and plasmid replicons were identified by searching against the databases from the Center for Genomic Epidemiology (http://www.genomicepidemiology.org/) (Bortolaia et al., 2020). The alignment of plasmids with similar structures was generated by BLAST Ring Image Generator (BRIG) (Alikhan et al., 2011) and Easyfig_win_2.1 (Sullivan et al., 2011).

### Construction of the Spectinomycin-Resistant pPM27_Vir Mutant

The spectinomycin-resistance-encoding gene was introduced into plasmid pPM27_Vir by an allelic exchange with a PCR-synthesized cassette encoding spectinomycin resistance *via* the lambda Red recombinase system (Datsenko and Wanner, 2000). First, plasmid pKD46 encoding the lambda Red recombinase system was electroporated into strain PM27. Simultaneously, the spectinomycin-resistance-encoding cassette flanked by the flippase recognition target (FRT) site sequence was amplified using primers (F: CACCGGCATATACCACCCGCTGGAAGGAGCTGCCGGTTGCCCGTTTCTGACAGGAAACAGCTATGAC and R: TGATCGGTAAAGTAAGCTCTGGCGGCAAGTCCCGTCTGTTATTTCGACGGGTAAAACGACGGCCAT), resulting in a PCR product flanked by regions homologous to 50-bp sequences upstream and downstream of a non-encoding region of plasmid pPM27_Vir. Then, the purified PCR product was electroporated into strain PM27 expressing the lambda Red recombinase system induced by 0.5% arabinose, followed by spectinomycin selection and temperature-induced curing of pKD46. The correct gene addition was verified by polymerase chain reaction (PCR).

### Conjugation Assay

Conjugation was performed using a spectinomycin-resistant PM27 mutant as a donor and a rifampin-resistant *E. coli* strain EC600 as a recipient. Both donor and recipient strains were cultured to the logarithmic phase (OD ~ 0.6) at 37°C in an LB medium. Then, 100 μl culture of the donor cells and 400 μl culture of the recipient cells were mixed and inoculated carefully on a 0.45-μm membrane, which was placed on an LB agar plate. After incubation at 37°C overnight, bacteria on the membrane were collected, resuspended in saline, and serially diluted. The diluted culture was spread on MacConkey agar plates containing 50 μg/ml spectinomycin and 600 μg/ml rifampin. The presence of the *rmpA* gene as a marker gene of virulence plasmid in transconjugants was determined by PCR. The successful transconjugant of *E. coli* strain EC600 was then treated as a donor and the *K. pneumoniae* strains as recipients to further determine the transferability of the virulence plasmid. MacConkey agar plates containing 50 μg/ml ampicillin and 50 μg/ml spectinomycin were used to select transconjugants. Conjugation was repeated one time to verify the reproducibility. Antimicrobial susceptibility testing was performed to differentiate between the donor and recipient strains. The introduced spectinomycin-resistance-encoding cassette in the *K. pneumoniae* strains was further excised by the plasmid pCP20, expressing the recombinase flippase (Flp) (Datsenko and Wanner, 2000). XbaI digestion and S1 nuclease pulsed-field gel electrophoresis (XbaI PFGE and S1-PFGE) were also performed to confirm the transfer of this plasmid through conjugation.

### Mucoviscosity Assay and Uronic Acid Quantification

The mucoviscosity of the capsule was determined using the sedimentation assay with modifications (Palacios et al., 2018). In brief, strains were cultured in LB broth at 37°C overnight. The cultures were normalized to an OD of 1.0/ml and centrifuged for 5 min at 1,000 *g*. The supernatant was gently removed without disturbing the pellet for OD600 measurement. Uronic acid was extracted and quantified as previously described with modifications (Palacios et al., 2018; Ernst et al., 2020). In brief, 500 μl of overnight culture for the mucoviscosity assay was mixed with 100 μl of 1% Zwittergent 3–14 in 100 mM citric acid and incubated at 50°C for 20 min. Cells were pelleted, and 300 μl of the supernatant was added to 1.2 ml of absolute ethanol, incubated at 4°C for 30 min, and centrifuged for 5 min at a maximum speed. The pellet was dried, resuspended in 200 μl of distilled water, and added to 1.2 ml of 12.5 mM sodium tetraborate in sulfuric acid, and the mixture was incubated for 5 min at 100°C and then on ice for 10 min. A 20-μl 0.15% 3-phenylphenol in 0.5% NaOH was added. After a 5-min incubation at room temperature, the absorbance at 520 nm was measured. The glucuronic acid content was determined from a standard curve of glucuronic acid and expressed as micrograms per OD unit. An ST23-K1 hypervirulent *K. pneumoniae* strain HvKP1088 and an ST11 CRKP strain FJ8 reported in our previous studies were included as controls for both assays (Zhang et al., 2016; Yang et al., 2019). The results were presented as the mean and standard deviation of data of three independent experiments. Unpaired two-sided Student's *t*-test was performed to analyze the statistical difference between the mucoviscosity and uronic acid levels of parental strains and transconjugants carrying the virulence plasmid using GraphPad Prism 7 (San Diego, CA, USA).

### Mouse Infection Model

A mouse bacteremia model was used to test the potential virulence of the *K. pneumoniae* strains. In this experiment, eight female ICR mice (4–5 weeks old, ~20 g) in each group were infected intraperitoneally with an inoculum of 1.0 × 10^3^ and 5.0 × 10^3^ CFUs of different strains of *K. pneumoniae*, respectively. The mortality rate of the test mice was observed and recorded for 1 week post-infection. Survival curves were generated using GraphPad Prism version 7.00. A statistical analysis was performed using the log-rank (Mantel–Cox) test recommended by Prism 7.00. An ST23-K1 hypervirulent *K. pneumoniae* strain HvKP1088 was used as a control for high virulence, while an ST11 CRKP strain FJ8 was used as a control for low virulence (Zhang et al., 2016; Yang et al., 2019). All animal experiments were approved by the Animal Research Ethics Sub-Committee, City University of Hong Kong. The animal experiments were repeated two times to assess the consistency of the data.

### Data Availability

Complete sequences of the chromosome of strain PM27 and plasmids pPM27_Vir and pPM27_2 have been deposited in the GenBank database under accession numbers CP076453–CP076455, respectively. Illumina and nanopore read data have been deposited in the GenBank database under BioProject PRJNA735573.

## Results

### Phenotypic and Genetic Characterization of Strain PM27

A *K. pneumoniae* strain PM27, which was identified by the Vitek 2 system (bioMérieux, France) and confirmed by the MALDI-TOF MS (Bruker, Germany), was recovered from a blood sample of a 63-year-old female patient in a hospital in Hong Kong in 2015. Stretching of the colonies on blood agar resulted in the formation of a string of ~6 mm in length. Antimicrobial susceptibility tests performed on strain PM27 showed that it was susceptible to all tested antibiotics, including β-lactams, aminoglycosides, ciprofloxacin, azithromycin, chloramphenicol, tetracyclines, and colistin (Table 1).

**Table 1:** Phenotypic and genotypic characteristics of the K. pneumoniae strain PM27 and its transconjugants.

**Strain ID**	**Bacterial species**	**ST type**	**MIC (μg/ml)** ^**a**^													***rmpA***	**Conjugation efficiency**
			**AMP**	**CAZ**	**CTX**	**MEM**	**GEN**	**AMK**	**AZI**	**CIP**	**CHL**	**TET**	**TIG**	**CLS**	**SPE**		
PM27	*K. pneumoniae*	66	8	0.5	0.06	0.06	0.5	1	2	0.008	4	2	0.5	2	16	+	NA
PM27-spe	*K. pneumoniae*	66	8	0.5	0.06	0.06	0.5	1	2	0.008	4	2	0.5	2	>128	+	NA
EC600	*E. coli*	NA	8	0.5	0.125	0.06	1	2	2	0.008	4	2	0.5	0.5	8	–	NA
EC600-TC	*E. coli*	NA	8	0.5	0.125	0.06	1	2	2	0.008	4	2	0.5	0.5	>128	+	1.25E-05
EH12PC	*K. pneumoniae*	23	64	0.25	0.06	0.06	0.5	1	4	0.008	2	2	0.5	2	8	–	NA
EH12PC-TC^b^	*K. pneumoniae*	23	64	0.25	0.06	0.06	0.5	1	4	0.008	2	2	0.5	2	>128	+	2.34E-08
PM8PC	*K. pneumoniae*	374	128	0.5	0.06	0.06	0.5	1	4	0.008	2	2	0.5	2	8	–	NA
PM8PC-TC	*K. pneumoniae*	374	128	0.5	0.06	0.06	0.5	1	4	0.008	2	2	0.5	2	>128	+	1.04E-08
14WZ-1	*K. pneumoniae*	11	>128	>128	>128	>128	0.5	1	4	>128	4	2	0.5	2	16	–	NA
14WZ-1-TC	*K. pneumoniae*	11	>128	>128	>128	>128	0.5	1	4	>128	4	2	0.5	2	>128	+	4.36E-08
25922	*E. coli*	NA	2	0.12	0.06	0.016	1	2	2	0.008	4	0.5	0.25	0.5	8	NA	NA

a *AMP, ampicillin; CAZ, ceftazidime; CTX, cefotaxime; MEM, meropenem; GEN, gentamicin; AMK, amikacin; AZI, azithromycin; CIP, ciprofloxacin; CHL, chloramphenicol; TET, tetracycline; TIG, tigecycline; CLS, colistin; SPE, spectinomycin. All tests were performed in duplicate, and each test included three biological replicates*.

b *Transconjugants EH12PC-TC, PM8PC-TC, and 14WZ-1-TC were not subjected to spectinomycin-resistance-encoding cassette excision by Flp*.

**Figure 1:**
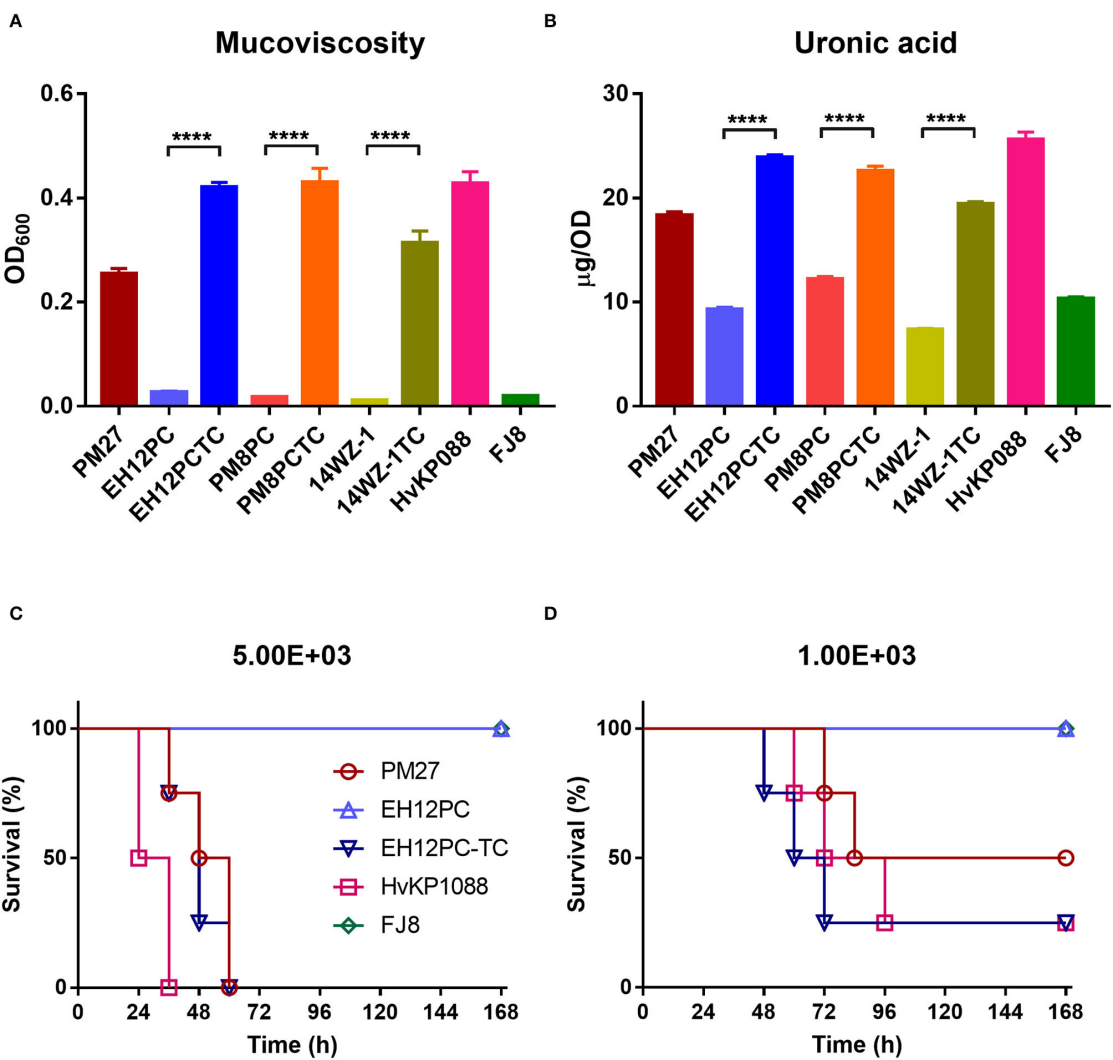
Virulence level of different bacterial strains. **(A)** Mucoviscosity and **(B)** uronic acid production for different *K. pneumoniae* strains. Each data point was repeated three times (*n* = 3). Data represent mean ± SEM. Unpaired two-sided Student's *t*-test was performed for strains EH12PC-TC vs. EH12PC (*P* < 0.0001), PM8PC-TC vs. PM8PC (*P* < 0.0001), and 14WZ-1-TC vs. 14WZ-1 (*P* < 0.0001). Survival of mice (*n* = 8) infected by 5 × 10^3^ CFU **(C)** and 1 × 10^3^ CFU **(D)** of each *K. pneumoniae* strain at 168 hrs. The test strains included the *K. pneumoniae* strain PM27, virulence plasmid-cured ST23-KL1 *K. pneumoniae* strain EH12PC, transconjugant strain EH12PC-TC, HvKP strain HvKP1088 (hypervirulence control), and the classic CRKP strain FJ8 (low-virulence control). The log-rank (Mantel–Cox) test was performed for curves of strains EH12PC and EH12PC-TC. A significant difference (*P* = 0.0069 and 0.0401 at an inoculum of 5 × 10^3^ and 1 × 10^3^ CFU, respectively) was observed between curves.

The capsule (CPS) of *K. pneumoniae* serves as one of the main virulence factors, and hypercapsule is associated with hypervirulence; thus, the hypermucoviscosity and CPS production of strain PM27 were determined to evaluate its virulence potential. The mucoviscosity of strain PM27 was determined using the sedimentation assay, and the CPS production was evaluated by quantifying the amount of uronic acid produced by strain PM27. The results showed that strain PM27 exhibited a high degree of mucoviscosity and uronic acid production, the levels of which were lower than the HvKP control strain ST23/K1 HvKP1088 but much higher than that of the low-virulence control strain FJ8 (Figures 1A,B). The virulence level of strain PM27 was further tested in a mouse infection model. Upon being infected for 1 week at an inoculum of 5 × 10^3^ CFU, the survival rate of mice was 0% with strain HvKP1088, 0% with strain PM27, and 100% with strain FJ8 (Figure 1C). In contrast, at a lower inoculum of 1 × 10^3^ CFU, the survival rate of mice was 25% with strain HvKP1088, 50% with strain PM27, and 100% with strain FJ8 (Figure 1D). These data all showed that the strain PM27 had a high virulence potential.

Strain PM27 was then subjected to whole-genome sequencing by both the Illumina NextSeq 500 platform and the long-read MinION platform to retrieve its complete genome sequences. The genome size of strain PM27 was 5,670,661 bp (base pair), including a 5.16-Mb chromosome and two plasmids with a size of 162,330 and 92,566 bp, respectively (Table 2). Strain PM27 was found to belong to ST66/KL2 type by Kleborate and Kaptive. Screening of resistance genes showed that this strain harbored the intrinsic *oqxAB* and *fosA* genes but did not harbor a *bla*_SHV_ gene. BLASTN against the virulence gene database showed that strain PM27 harbored a number of virulence genes, including type 3 fimbriae-encoding gene *mrkABCDFHIJ*, yersiniabactin- and colibactin-encoding genes, a regulator of mucoid phenotype gene *rmpA*, salmochelin-encoding gene *iroBCDN*, and aerobactin-encoding gene *iucABCDiutA* (Table 2). The salmochelin and aerobactin lineages were predicted as *iro* 2 and *iuc* 2, respectively, and *rmpA* was determined as a variant *rmpA_9* (KpVP-2) according to Kleborate.

**Table 2:** Genetic characterization of the *K. pneumoniae* strain PM27.

**Genetic element**	**Size (bp)**	**Virulence genes**	**Resistance genes**	**Inc type**	***tra* gene**
Chromosome	5,415,765	*mrk, ybt, clb*	*oqxAB, fosA*	NA	NA
pPM27_Vir	162,330	*rmpA, iro, iuc*	–	FIB_K_	+
pPM27_2	92,566	–	–	FIA(HI1)	+

**Figure 2:**
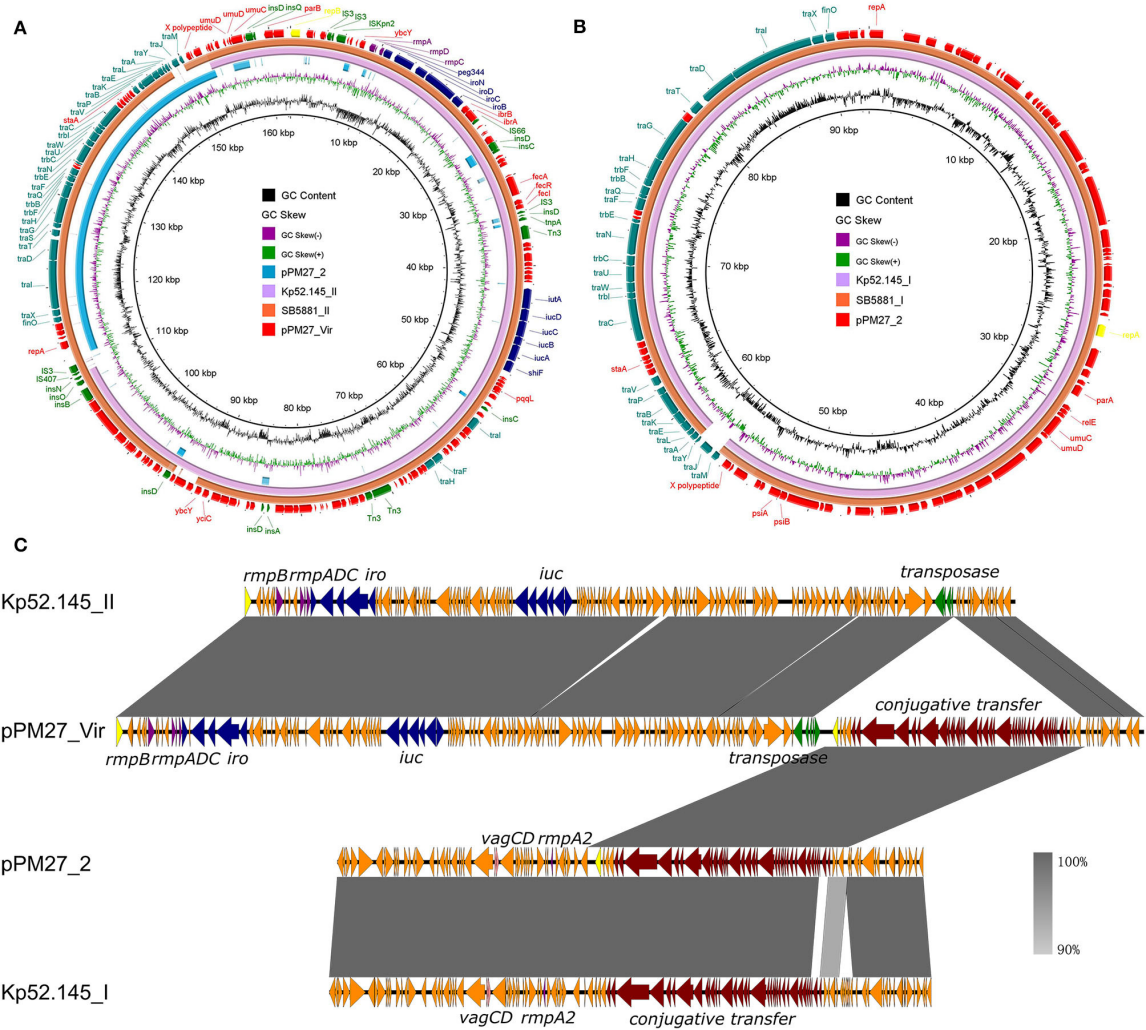
Alignment of plasmids pPM27_Vir and pPM27_2 with similar plasmids. **(A)** Alignment of plasmid pPM27_Vir with similar plasmids by BRIG. Plasmid pPM27_Vir showed the highest similarity to SB5881 plasmid II (GenBank accession no. LR792629.1, 97% coverage and 99.89% identity) and Kp52.145 plasmid II (GenBank accession no. FO834905.1, 73% coverage and 99.97% identity). **(B)** Alignment of plasmid pPM27_2 with similar plasmids by BRIG. Plasmid pPM27_2 showed the highest similarity (98% coverage and 99.9% identity) to both plasmids, namely, Kp52.145 plasmid I (GenBank accession no. FO384904.1) and SB5881 plasmid I (GenBank accession no. LR792630.1). **(C)** Alignment of plasmids pPM27_Vir and pPM27_2 with Kp52.145 plasmids by Easyfig. Plasmid pPM27_Vir could have been originated from Kp52.145 plasmid II by fusion with the IncF plasmid conjugative transfer genes from plasmid pPM27_2.

The assembly results showed that strain PM27 carried two distinct plasmids with a size of 162,330 and 92,566 bp, respectively, designated as pPM27_Vir and pPM27_2 (Table 2). The virulence genes, namely, *rmpA, iroBCDN*, and *iucABCDiutA*, were found to be located in the 162,330-bp plasmid pPM27_Vir. The plasmid pPM27_Vir was found to belong to the IncFIB_K_ type with a GC content of 49.77% and comprised 188 predicted protein-coding genes. Using BLAST against the NCBI database, this plasmid exhibited the highest similarity (97% coverage and 99.89% identity) to SB5881 plasmid II (GenBank accession no. LR792629.1), a 160,760-bp plasmid recovered from an ST66-K2 *K. pneumoniae* strain isolated in France (Figure 2A). Interestingly, plasmid pPM27_Vir showed 73% coverage and 99.97% identity to Kp52.145 plasmid II (GenBank accession no. FO834905.1), a distinct non-self-transmissible KpVP-2-type virulence plasmid carrying the *iuc2*/*iro2* lineages and *rmpA*. The inserted DNA fragment in plasmid pPM27_Vir was the IncF plasmid conjugative transfer genes from plasmid pPM27_2 (Figure 2A). Plasmid pPM27_2 was found to belong to the IncFIA(HI1) type with a GC content of 49.85% and comprise 108 predicted protein-coding genes. Plasmid pPM27_2 showed the highest similarity (98% coverage and 99.9% identity) to both plasmids, namely, Kp52.145 plasmid I (GenBank accession no. FO384904.1) and SB5881 plasmid I (GenBank accession no. LR792630.1) (Figure 2B). The alignment of plasmids pPM27_Vir and pPM27_2 with Kp52.145 plasmids indicated that pPM27_Vir may originate from Kp52.145 plasmid II by fusion with the IncF plasmid conjugative transfer genes from plasmid pPM27_2 (Figure 2C). Further analysis identified a 1,222-bp region encoding insertion sequence IS*407* at the upstream of the insertion and a 219-bp homologous region at the downstream. The 1,222-bp IS*407* was found to show 100% coverage and identity to a region from the chromosome of strain PM27. The 219-bp homologous non-coding region showed 95% identity between plasmids pPM27_Vir and pPM27_2. Thus, it was surmised that the IS*407* from the chromosome targeted pPM27_Vir and mediated fusion of the conjugative transfer region from pPM27_2 into it. The circulation then took place by the downstream homologous region, resulting in the generation of the mosaic plasmid pPM27_Vir.

### Conjugation of the Virulence Plasmid pPM27_Vir

**Figure 3:**
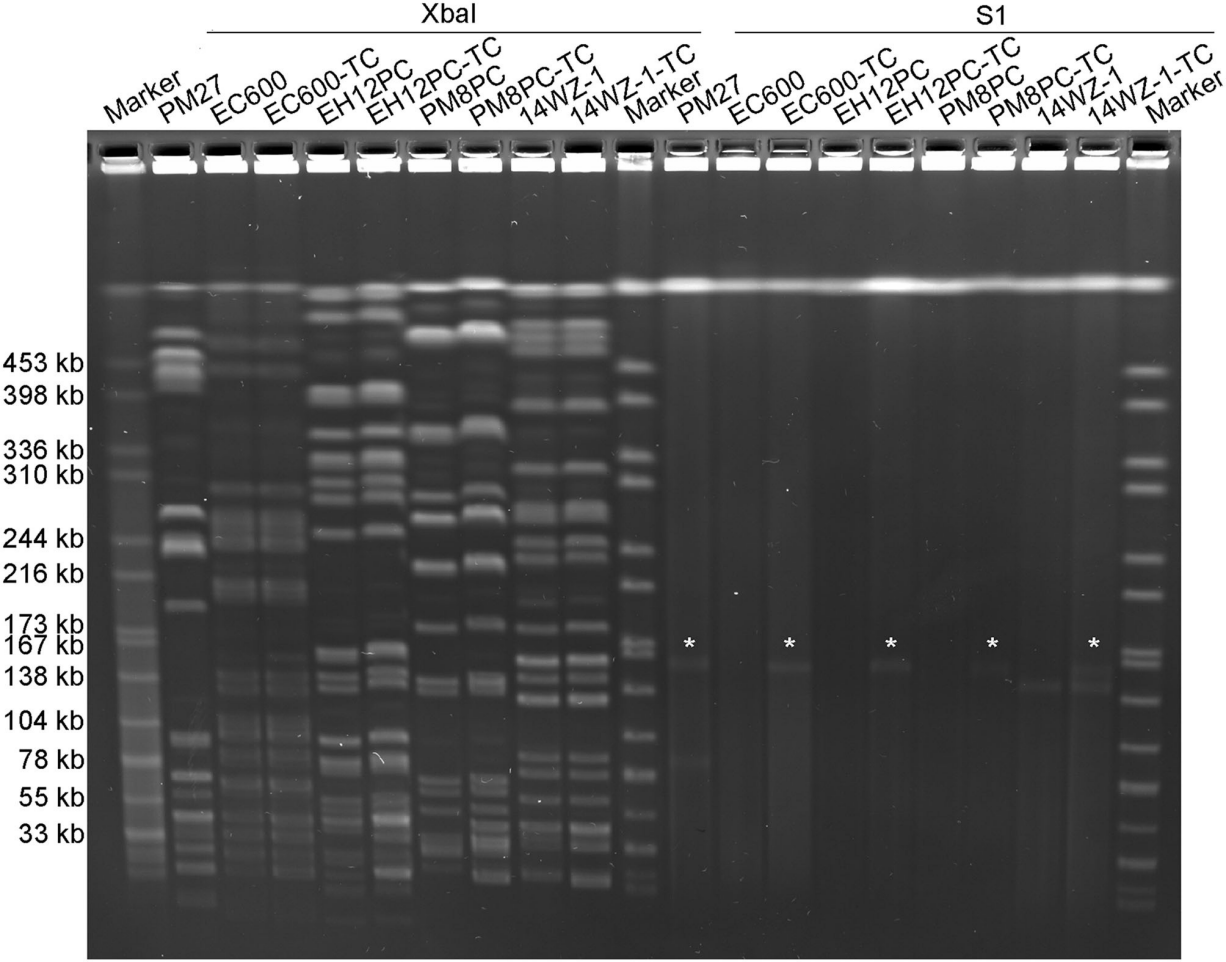
XbaI PFGE and S1-PFGE analyses of the *K. pneumoniae* strain PM27 and its transconjugants. XbaI PFGE and S1-PFGE were repeated two times for all strains with the same results. “*” is denoted for virulence plasmid pPM27_Vir.

The potential of plasmid pPM27_Vir to be conjugative was then determined. As there were no selection markers, such as antibiotic- or heavy metal resistance-encoding genes in plasmid pPM27_Vir, we first introduced a spectinomycin resistance gene into plasmid pPM27_Vir using the lambda Red recombinase system without disrupting any genes, resulting in the virulence plasmid mutant pPM27_Vir-spe. The spectinomycin-resistant PM27 mutant, PM27-spe, was then treated as a donor and the *E. coli* strain EC600 as a recipient for the conjugation experiment. Transconjugant EC600-TC exhibited similar antimicrobial susceptibility as the recipient strain EC600 except for additional resistance to spectinomycin, and it was positive for the *rmpA* gene (Table 1). PFGE results indicated identical XbaI digestion profiles of transconjugant EC600-TC to strain EC600 and acquisition of a 160-kbp plasmid by S1-PFGE (Figure 3). The results indicated that plasmid pPM27_Vir-spe could be readily transferred to strain EC600 at an efficiency of 1.25E-05 (Table 1, Figure 3). We further tested the transferability of plasmid pPM27_Vir-spe from strain EC600 to *K. pneumoniae* strains of various types. We used a virulence plasmid-cured ST23-KL1 strain EH12PC, a virulence plasmid-cured ST374-KL2 strain PM8PC, and an ST11-KL47 CRKP strain 14WZ-1 as recipients. The results showed that plasmid pPM27_Vir-spe could be transferred to all these three strains at an efficiency range from 1.04E−08 to 4.36E−08 (Table 1, Figure 3). Transconjugants EH12PC-TC, PM8PC-TC, and 14WZ-1-TC exhibited similar profiles as each recipient except for additional 160-kbp plasmid and resistance to spectinomycin.

### Contribution of Virulence Plasmid pPM27_Vir in *K. pneumoniae* Strains

To determine whether the acquisition of virulence plasmid pPM27_Vir could cause a significant increase in the virulence level of *K. pneumoniae*, all *K. pneumoniae* transconjugants were subjected to hypermucoviscosity and uronic acid quantitative assays, following Flp excision of the spectinomycin-resistance-encoding cassette. The results showed that all *K. pneumoniae* transconjugants exhibited a significant increase in the mucoviscosity compared with the parental strains: EH12PC-TC vs. EH12PC (*P* < 0.0001), PM8PC-TC vs. PM8PC (*P* < 0.0001), and 14WZ-1-TC vs. 14WZ-1 (*P* < 0.0001). Similarly, uronic acid production of all the transconjugants was increased significantly compared with the parental strains: EH12PC-TC vs. EH12PC (*P* < 0.0001), PM8PC-TC vs. PM8PC (*P* < 0.0001), and 14WZ-1-TC vs. 14WZ-1 (*P* < 0.0001). A virulence potential of strains EH12PC and EH12PC-TC was further tested using the mice infection model. Upon being infected for 1 week at an inoculum of 5 × 10^3^ CFU, the survival rate of mice was 0% with strain EH12PC-TC and 100% with strain EH12PC with a significant difference (*P* = 0.0069) (Figure 1C). In contrast, at a lower inoculum of 1 × 10^3^ CFU, the survival rate of mice was 25% with strain EH12PC-TC and 100% with strain EH12PC with a significant difference (*P* = 0.0401) (Figure 1D).

## Discussion

The virulence of *K. pneumoniae* is highly associated with two dominant types of virulence plasmids, namely, KpVP-1 and KpVP-2 (Lam et al., 2018). The virulence-associated loci encoding siderophores aerobactin and salmochelin as well as the regulator of mucoid phenotype RmpA are highly conserved between these two types of virulence plasmids (Wu et al., 2009; Lery et al., 2014). The hypervirulent ST66-K2 strain Kp52.145 is one of the widely studied strains that carried KpVP-2 (Nassif and Sansonetti, 1986). This virulence plasmid was demonstrated to contribute to hypervirulence in *K. pneumoniae* strains (Nassif and Sansonetti, 1986). Since its emergence, ST66-K2 HvKP carrying KpVP-2 has been reported to cause various human infections, including endogenous endophthalmitis, tonsillopharyngitis, acute otitis media, meningitis, bacteraemia, and bacteriuria worldwide (Rodrigues et al., 2020; Kamau et al., 2021; Klaper et al., 2021). These strains exhibited a few SNPs, indicating clonal expansion of this lineage (Rodrigues et al., 2020). Both KpVP-1 and KpVP-2 are considered to be non-conjugative due to the lack of conjugation machineries. Recent work has indicated that KpVP-1 can be mobilized by the help of other conjugative plasmids (Xu et al., 2021) and also by generating mosaic plasmids (Yang et al., 2019). In contrast, the transmission of KpVP-2-type virulence plasmids has been rarely reported, and the mechanisms underlying the dissemination of this kind of plasmid remained unknown. In this study, we characterized a KpVP-2-type virulence plasmid from a clinical virulent *K. pneumoniae* strain PM27. This strain also belonged to the ST66-KL2 lineage and exhibited high virulence in a mice infection model. Two plasmids were identified in strain PM27, including an IncFIB_K_ virulence plasmid and an IncFIA plasmid. The virulence plasmid pPM27_Vir was generated by the integration of the conjugative transfer genes from plasmid pPM27_2 into the KpVP-2-type plasmid Kp52.145 II. A similar plasmid has been reported in the ST66-K2 strain SB5881 that caused community-acquired infections in France (Rodrigues et al., 2020). Kp52.145 plasmid II is non-conjugative, whereas plasmid pPM27_Vir might be conjugative after acquiring the conjugative transfer genes, promoting the transmission of these virulence factors among *K. pneumoniae* strains. The transferability of plasmid pPM27_Vir was then determined, and it was readily conjugated to the *E. coli* strain EC600 and the *K. pneumoniae* strains of various types, which are commonly existing clinically. Furthermore, acquisition of this plasmid resulted in significant increases in mucoviscosity and CPS production of *K. pneumoniae* strains and an increase in virulence potential of a KL1 *K. pneumoniae* strain EH12PC in mice. The transferability of plasmid pPM27_Vir provides the possibility of dissemination of this kind of virulence plasmid. However, the real scenario is complicated and monitoring of this kind of plasmid in pathogens in local hospitals and worldwide is required to understand the real dissemination situations. In conclusion, the generation of this kind of conjugative virulence plasmid may promote the transmission of virulence determinants in the *K. pneumoniae* strains. Of particular concern is the generation of plasmids that confer both virulence and resistance, which may make this pathogen more difficult to treat.

## Data Availability Statement

The data presented in the study are deposited in the GenBank database, BioProject accession number PRJNA735573.

## Ethics Statement

The animal study was reviewed and approved by Animal Research Ethics Sub-Committee, City University of Hong Kong.

## Author Contributions

XY performed the experiment and drafted the manuscript. XL performed the experiment. YX and CY helped with the sequencing and conjugation experiment. EC edited the manuscript and contributed to the experimental design. HS provided the clinical strains of *K. pneumoniae*. SC designed and supervised the study and interpreted the data. All authors contributed to the article and approved the submitted version.

## Funding

The research was supported by Guangdong Major Project of Basic and Applied Basic Research (2020B0301030005) and the NSFC/RGC grant (NSFC-RGC, N_PolyU521/18) from the National Natural Science Fund in China and the Research Grant Council of the Government of Hong Kong SAR.

## Conflict of Interest

The authors declare that the research was conducted in the absence of any commercial or financial relationships that could be construed as a potential conflictof interest.

## Publisher's Note

All claims expressed in this article are solely those of the authors and do not necessarily represent those of their affiliated organizations, or those of the publisher, the editors and the reviewers. Any product that may be evaluated in this article, or claim that may be made by its manufacturer, is not guaranteed or endorsed by the publisher.
